# Emergency management and resuscitation of poisoned patients: perspectives from "down under"

**DOI:** 10.1186/1757-7241-17-36

**Published:** 2009-08-23

**Authors:** Mark Little

**Affiliations:** 1 Royal Perth Hospital, Welllington St, Perth, Western Australia, Australia; 2 University of Western Australia, Crawley WA 6009, Perth, Australia

## Introduction

Deliberate self poisoning (DSP) is a common presentation to an emergency department (ED), being an acute medical exacerbation of a chronic psychosocial disorder [1]. At Sir Charles Gairdner Hospital (SCGH) in Perth Western Australia, DSP and intoxication accounts for 4.6% of all ED presentations [2], the Austin Hospital in Melbourne, reported 650 presentations per year (2% of their ED presentations) [3]. In the UK, Kappur estimated an annual rate of presentation to a UK hospital of 310/100000 population and estimated 170 000 presentations to EDs in the UK [4]. One American study estimated an annual rate of ED presentation of self harm in 7 – 24 years old at 225.3 per 100 000 population [5].

With such a caseload it is important that there is a structured process to the management and disposition of cases, as Boyle and her colleagues have described in their review article on the management of the critically poisoned patient [6]. As Boyle discussed resuscitation is an essential part of the management of the poisoned patient, and often this and good supportive care is all that is required in the patients management.

## Indications for intubation

The decision to intubate a poisoned patient with a Glasgow Coma Score (GCS) of 9–13 remains a difficult dilemma. In an Australian study of over 4500 overdose admissions there was a prolonged increase in ICU length of stay for a patient with aspiration (126 hrs for those who aspirated vs 14.7 hrs for those who did not) and death (8.5% vs 0.4%) [7]. I therefore advocate early intubation with DSP in patients developing a reduced LOC

## Controversies in cardiopulmonary resuscitation

Cardiac arrest in a poisoned patient is another area where the toxicological management will be different to standard ED protocols. Most cardiac arrests in adult ED's are due to ischaemic heart disease and usually there is poor success after a period of time (eg 30 – 60 m) of resuscitation. In a poisoned patient, patients are often young and if they are supported through this period they will totally recover. I advocate prolonged CPR (my colleague teaches our junior staff to continue until at least the end of their shift!) and there may be roles for antidotes (such as a digoxin fragment antibody, bicarbonate and high dose insulin) and heroic measures such as cardiopulmonary bypass. For example, in a series of 56 cardiac arrests due to digoxin toxicity the survival with the use of digoxin fragment antibody was 54%, making it the most successful intervention in cardiac arrest [8]. Mortality prior to the use of digoxin fragment antibody was 100%. In the 2005 American Heart Association Guidelines for cardiopulmonary resuscitation and emergency cardiovascular care, there is a separate section specifically on toxicology in emergency cardiovascular care [9], which many clinicians may be unaware of.

## Management issues

In Australia, after resuscitation, our approach is to then perform a risk assessment to identify the severity (or otherwise) of the poisoning based on a variety of factors included the agent and dose ingested, the time since ingestion, symptoms and signs exhibited and any premorbid factors. This risk assessment would dictate further management [1].

As Boyle has described there are many issues to be considered [6]. Hyperthermia is an important sign not to be missed. In Australia due to the increased usage the increased availability of serotonergically active drugs (including amphetamines) we are commonly seeing serotonin syndrome in our patients, this being a clinical diagnosis. Lower limb clonus is highly suggestive of the diagnosis [10], and its diagnosis and management is well detailed by Boyle's article.

## Toxicology in Australia

In Australian there has been the establishment of toxicology services managing poisoned patients. In Perth, the Western Australian Toxicology Service (WATS) runs an emergency department based toxicology service across three tertiary hospitals. Emergency Physicians with 2 year subspecialty training in toxicology admit patients directly under their care to the intensive care unit or the emergency observation unit (EOU). Working closely with psychiatric, drug and alcohol and social worker services care is provided in parallel with poisoned patients in both the ICU and EOU [1,2,11]. Patients are returned to the EOU as soon as a patient is cleared from the ICU. In 2005 SCGH (one of the three hospitals) had 1859 DSP presentations and 1010 admission with 85 ICU admissions. The average length of stay was 12 hours. In the first 12 months of establishing a toxicology service in Melbourne, Lee et demonstrated a significant reduction in length of stay for poisoned patients admitted to their service. Length of stay (LOS) for uncomplicated admissions dropped from 1.97 days to 1.4 days, and for complicated admissions the LOS dropped from 5.59 days to 1.92 days [3].

In Perth, our commonest toxicological presentation is an overdose due to alcohol and benzodiazepines. Other commonly overdosed agents are the SSRI and SNRI's, atypical antipsychotics, inparticularly quetiapine and paracetomol (although we would use N acetyl cysteine only once a fortnight). Quetiapine would be our commonest agent causing coma requiring ventilation and admission to the ICU. Until the last 6 months presentations due to amphetamine intoxication was about 1% of all ED presentations to Royal Perth Hospital, but in recent times opiate overdoses have increased. Tricyclic antidepressant overdoses are rarely seen nowadays in Perth.

## Envenomings

Contrary to the public perceptions, snakebite is rarely seen. We would admit about 5 envenomed patients per year to Royal Perth Hospital, although many other non envenomed snakebites are seen. Around Perth the most common envenoming is due to the dugite snake (*Pseudonaja affinis*) and patients present with a venom induced consumptive coagulopathy. Occasionally these patients have sudden cardiovascular collapse, but often they present with non specific symptoms and incoagulable blood. Earlier this year one of our patients died from an intracerebral haemorrhage, however death is rare. There is a monovalent antivenom that we use to treat these patients. In recent times we have described a rare complication of microangiopathic haemolytic anaemia (MAHA) developing after envenoming by snakes of the *Pseudonaja *genus [12]. Even if these patients are envenomed, unless they require ventilation, all are managed in our EOU.

More commonly during summer, we manage many patients envenomed by the red back spider (*Lactrodectus hasselti*). We have one of the highest incidences in the world of the syndrome of lactrodectism. Patients present with significant bite site pain or systemic features of generalised pain, autonomic features including sweating hypertension and tachycardia. No deaths have occurred in Australia since the antivenom was introduced in the 1950's, and in Australia more red back spider antivenom is administered than all the other snake, spider, stonefish, box jellyfish antivenoms available.

## Toxicology network in Australia

In Australia we have a Poisons Information Centre (PIC) network that utilises one national phone number. There are PICs in Perth, Sydney, Brisbane and Melbourne and we have a system so that one centre (usually NSW) takes all national calls overnight. In 2008, there were over 235 000 calls to this network [11]. Paracetomol calls remain the commonest call referred to clinical toxicologists. In Australia clinical toxicology is a new speciality there are about 20 clinical toxicologists and 6 fellows training. There are a number of emergency medicine trainees with 6 month accredited training posts in clinical toxicology. As well as running clinical toxicology services around the country, clinical toxicologists provide back up to the PIC network, and meet 5 times a year in Sydney to discuss interesting cases and management issues. All calls taken by the clinical toxicologists are peer reviewed by having calls emailed to all PIC staff. Locally WATS meets each week, reviewing cases and leading teaching sessions for emergency medicine staff at a number of hospitals in Perth. We have also published Australasia's first textbook in clinical toxicology which is being used at most Emergency departments around the country [13].

## Conclusion

In conclusion, DSP is a common presentation to an ED. Boyle and her colleagues are to be commended for their article detailing a structured approach to the poisoned patient. In my service, the use of the EOU in the care of the poisoned patient had dramatically improved care and reduced length of stay. With a structured approach to these patients and dedicated toxicology units for these patients I believe quality care will improve.

## Competing interests

The author declares that they have no competing interests.
